# Carcinome adénoïde kystique des paupières: à propos d'un cas

**DOI:** 10.11604/pamj.2014.17.239.3488

**Published:** 2014-03-28

**Authors:** Hakima Elouarradi, Moulay Zahid Bencherif

**Affiliations:** 1 Université Mohammed V Souissi, Service d'Ophtalmologie A de l'hôpital des spécialités, Centre hospitalier universitaire, Rabat, Maroc

**Keywords:** Carcinome adénoïde kystique, paupières, Cystic adenoid carcinoma, eyelids

## Image en medicine

Il s'agit d'une femme âgée de 68 ans, sans antécédents particuliers, présentant une tuméfaction de la paupière supérieure droite (A) remontant à plus d'un an et dont la biopsie avait objectivé un carcinome; la patiente a été reprise un mois après pour résection complète de la tumeur (B, C) et l'examen histopathologique a conclut à un carcinome adénoïde kystique (D) probablement sur adénome pléomorphe. On n'a observé aucune récidive pendant les 6 mois suivant le traitement chirurgical. Le carcinome adénoïde kystique de la paupière est une tumeur rare touchant les sujets d’âge moyen. C'est une tumeur fréquente des glandes salivaires, et également décrite au niveau de l'utérus, du sein, du conduit auditif externe et des bronches. Sa localisation palpébrale est exceptionnelle, pouvant dériver des glandes de Moll et des glandes lacrymales accessoires de la conjonctive. Cliniquement, la lésion se présente comme un nodule ou un placard infiltré, indolore, à développement lent. L’évolution est marquée par des récidives fréquentes. Les principaux diagnostics différentiels sont le carcinome basocellulaire de type adénoïde, et le carcinome syringomateux. Le traitement consiste en l'exérèse complète de la tumeur avec la vérification des marges de résection. Le pronostic est favorable à condition d'un diagnostic précoce et d'un traitement efficace.

**Figure 1:**
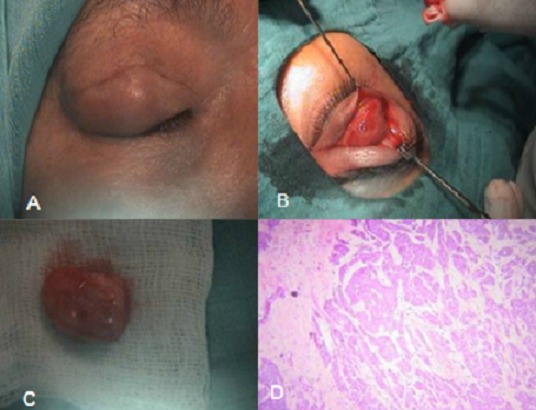
A) Aspect préopératoire de la masse palpébrale, vue de face; B) Exploration chirurgicale de la tumeur; C) Aspect macroscopique de la tumeur; D) Aspect histologique de la tumeur

